# Distributed optical fibre sensing for early detection of shallow landslides triggering

**DOI:** 10.1038/s41598-017-12610-1

**Published:** 2017-10-31

**Authors:** Luca Schenato, Luca Palmieri, Matteo Camporese, Silvia Bersan, Simonetta Cola, Alessandro Pasuto, Andrea Galtarossa, Paolo Salandin, Paolo Simonini

**Affiliations:** 1 National Research Council, Research Institute for Geo-Hydrological Protection, Padova, 35127 Italy; 2 University of Padova, Dept. of Information Engineering, Padova, 35131 Italy; 3 University of Padova, Dept. of Civil, Environmental and Architectural Engineering, Padova, 35131 Italy

## Abstract

A distributed optical fibre sensing system is used to measure landslide-induced strains on an optical fibre buried in a large scale physical model of a slope. The fibre sensing cable is deployed at the predefined failure surface and interrogated by means of optical frequency domain reflectometry. The strain evolution is measured with centimetre spatial resolution until the occurrence of the slope failure. Standard legacy sensors measuring soil moisture and pore water pressure are installed at different depths and positions along the slope for comparison and validation. The evolution of the strain field is related to landslide dynamics with unprecedented resolution and insight. In fact, the results of the experiment clearly identify several phases within the evolution of the landslide and show that optical fibres can detect precursory signs of failure well before the collapse, paving the way for the development of more effective early warning systems.

## Introduction

Shallow landslides are known to be a severe threat to people and structures, especially in the case of some flow-like landslides^1^, which travel at significant rates for long distances. Starting from the detaching area, flowslides erode and engage large amounts of soil along their path and finally discharge a great impact energy to engineering structures^2^.

Over the past decades, significant efforts have been undertaken both to understand the mechanisms responsible for landslide triggering better, as well as to identify precursors of instability suitable for detection^3,4^. In this way, effective early warning systems can be implemented. All the triggering factors, e.g., slope saturation and groundwater seepage, excavation, erosional processes or seismic action, typically cause an increase in shear stresses and/or pore water pressures. This increase reduces the safety factor *FS*, defined as the ratio between the resistant forces (i.e. the resistance of the soil *τ*_*max*_) and the driving ones (i.e. the mobilised shear stress *τ*_*mob*_). As a consequence of that, large shear strains occur in the sliding mass, tending to form, in most cases, a single or multiple sliding surfaces. When *FS* approaches the unit value, the shear strain rate exponentially increases, up to infinite. At the same time, instability becomes irreversible. The strain is therefore undoubtedly one of the most important physical parameters in landslide monitoring^5^.

In the recent years, several fibre optic sensors (FOSs) have been proposed for the measurement of strain in geotechnical applications, including landslide monitoring^6–8^. Most of the proposed sensors are single-point, mimicking existing traditional probes such as inclinometers^9^ and extensometers^10^. More than ten years ago, at the Public Works Research Institute in Japan, Brillouin optical time domain reflectometry (BOTDR) was investigated for landslide monitoring, highlighting important issues regarding measured strain vs. actual displacement^11^. Few years later, optical time domain reflectometry (OTDR) was introduced as a distributed sensing technology for the detection of soil displacement at the ground surface^12^. OTDR, however, provided very limited spatial accuracy. In the same period, other authors focused on Brillouin optical time domain analysis (BOTDA), achieving a spatial resolution on the meter scale, which is sufficient to determine landslide boundaries^6,13^.

Despite the efforts made in the last ten years or more, the correlation between the dynamics of landslides and the strain measured by optical fibre sensors is still not completely clear^14^. Recently, the results of proof-of-concept pullout tests suggest that the optical fibres could be successfully used for measuring strain in the soil since it seems to be possible to effectively couple the fibre cable with the soil^15,16^. Of course, this coupling is largely dependent on the frictional contact at the interface cable-soil but also on the accumulated relative displacement.

In real slopes, the lack of control of the measurement conditions represents a strong hindrance in obtaining significant and representative measurements. This has led some authors to test such sensors on large-scale, yet controllable, physical models^8,17–19^. In this respect, one of the advantages of fibre optic sensors is their negligible invasiveness so that the sensors do not interfere with the phenomenon they are designated to monitor. Recently, it has been proved that distributed fibre optic sensors have great potential as an entirely new tool for monitoring early deformations in small scale physical models^8,18,20^. As a proof of concept, a BOTDA scheme was used to measure the strain induced in two spans of simple tight-buffered cable, with a spatial resolution of some tens of centimetres inside a 1.35 × 0.5 × 0.1 m flume of volcanic ash.

When dealing with physical models, one of the main constraints is the reduced scale, which requires very high spatial resolution of the measurement system^21^. In fact, spatial sampling in the order of tens of centimetres is not optimal for physical models of few meters or less.

The first goal of this research is to present and discuss the results of an experimental investigation carried out in a large-scale physical model. The investigation evaluates the applicability of distributed fibre optic sensors for understanding the early-stage evolution of rainfall-induced slope instability. The second goal is to effectively measure and interpret the evolving strains as triggering signals of landslide occurrence and their dependence on the increasing slope saturation. To assure high resolution, a very densely distributed optical fibre strain sensing system, with centimetre spatial resolution, was applied to the model. For the first time in a large-scale model, a very detailed and informative map of the strain on the failure surface was thus obtained, as well as a clearer understanding of the failure mechanisms.

## Methods

**Figure 1:**
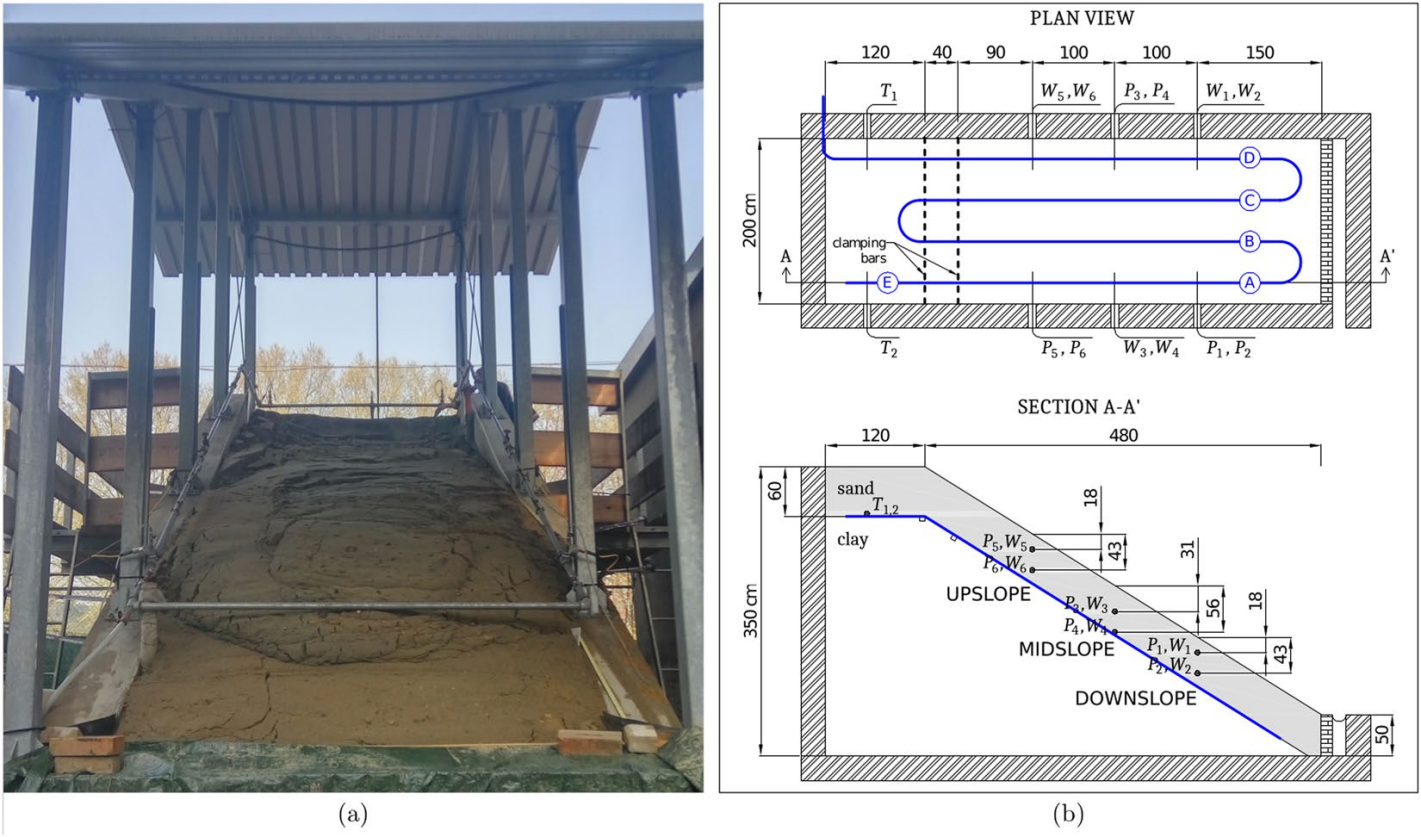
(**a**) The andslide occurred during the test in the large-scale physical model. (**b**) Top and lateral view of the instrumented flume; vertical dashed lines in the top view correspond to the clamping bars. The meandering optical fibre cable is represented in blue. Capital letters A,B…E identify spans of the fibre under measurement; ${P}_{1},\ldots {P}_{6}$ are pore water pressure sensors (i.e. tensiometers), ${W}_{1},\ldots {W}_{6}$ and ${T}_{1},{T}_{2}$ correspond to volumetric water content and temperature probes, respectively. The solid grey area in the lateral view represents the uniform fine sand layer; positions and depths of hydrological sensors, cable and bars are also indicated.

The experiment was carried out in a large-scale physical model of a slope built inside a 6 × 2 m reinforced concrete structure (see Fig. 1)^22^. The maximum height of the lateral walls measures 3.5 m and decreases linearly to 0.5 m to have a slope of approximately 32°. Several holes pass through the side walls to allow the insertion of conventional sensors and probes from the sides. At the slope toe, a draining brick wall retains the slope, allowing water to discharge. To reproduce the physics and dynamics of shallow landslides with a pre-defined failure surface, a constant thickness layer of permeable soil, made of fine non-compacted sand (in a relatively loose state) was deployed on top of a slope made of a less permeable, well-compacted, sandy clay. The top layer, prone to collapse, is 60 cm thick, while the entire slope is about 5 m long. A geotechnical characterisation of the soils used in the test is reported in the *Supplementary information*.

In order to drive this layered slope to collapse, rainfall was artificially produced by a system of nozzles located on the supports of the structure’s roof. This system was engineered to generate a relatively uniform rainfall intensity with drops of a small diameter to avoid surface erosion^23^. During the rainfall, water infiltrated in the shallow fine sand, reaching the less permeable sandy clay, thus saturating the upper layer. The increase in pore pressures triggered the slope collapse at the interface between the sand layer and the sandy clay bed.

The model has been equipped with a BRUsens© strain v9 cable (Brugg Kabel AG), embedded into the soil between the two layers mentioned above. The measurement interval of the cable is within 1% of strain, which is typically achieved with a tensile load of 470 N. The sensing fibre is hermetically sealed in a metal tube that provides protection and stiffness to the cable. To enhance the grip with the soil, the cable is coated with a plastic sheath corrugated with small indentations (0.7 mm deep and 5 mm spaced). The cable was preventively characterised to verify the suitability of its mechanical coupling with the soil. This was achieved by measuring the strain exerted on the cable in pullout tests. The details and results of these tests are reported in the *Supplementary information* together with some additional information on the cable structure.

The arrangement of the sensing cable is shown in the upper plot of Fig. 1(b). The cable, about 35 m in length, is deployed along four main straight spans running longitudinally down the slope at a distance of 0.25, 0.75, 1.25 and 1.75 m from the left lateral wall, respectively. The straight spans, labelled in the figure with capital letters A, B, C and D, are mechanically clamped onto two aluminium profiles attached to the lateral walls, at the top of the slope. These are represented by vertical dashed lines in the figure and sit at the layers’ interface, with the upper profile at the top of the slope and the lower one 0.4 m down the slope. The cable is unconstrained at the toe. Only strain exerted on the fibre along the four straight spans, between the upper bar and the toe, was measured and analysed. As described below, further measurements were taken along span E (continuation of span A) to compensate data for the effects of temperature variations.

An optical frequency domain reflectometer^24^ (OBR4600 from Luna Innovation Inc.) was used to measure the strain exerted on the cable by the landslide with a spatial resolution of 10 mm. The device measures the backreflected Rayleigh shift caused by the strain and, using a conversion factor, the shift is transformed into local strain variation (see *Supplementary information* for further details). As for fibre Bragg gratings, the reflected spectrum depends on strain and temperature variations. Inferring one of the two parameters from the optical data requires the other to be either constant or, by other means, known during the test. In this regard, it is important to note that, during the experiment, the air temperature varied negligibly and direct sunlight was carefully screened. Therefore, the only possible source of temperature variation in the experiment was the water. Since the rainfall-induced infiltration was relatively uniform, we used the spectral shift measured within the strain-free span E as a measure of water-induced temperature variation.

In addition, other conventional electric sensors were installed in the artificial slope (see Fig. 1(b)). Specifically, three pairs of tensiometers (${P}_{1},\ldots {P}_{6}$), measuring pore water pressure were located at different depths in upslope, mid-slope and downslope positions, at approx. 25 cm away from the lateral walls. The mid-slope sensors were located at depths of approximately 31 and 56 cm, while the upslope and downslope sensors at depths of about 18 and 43 cm, respectively.

Three additional pairs of water content reflectometer probes (${W}_{1},\ldots {W}_{6}$), measuring volumetric soil moisture^25^, were installed at mirror positions with respect to the tensiometers, to collect pairs of water content/pressure head data at different depths along the slope.

Moreover, two temperature probes (*T*_1_ and *T*_2_) were installed at the same depth (56 cm) on the two sides of the slope in the top terrace, with the aim of monitoring the evolution of soil temperature close to the interface between the two soil layers. Finally, two tipping-bucket flow gauges were also available, with the purpose of continuously monitoring the surface and subsurface components of the slope outflow.

The landslide experiment was performed by applying an average rainfall intensity of 160 mm/h, which determined the same reduced surface erosion that is attained with a natural rainfall of 10 mm/h^23^. Overall, the test lasted 137 min, from when the rainfall started to when the slope collapsed. For most of the experiment, the landslide body did not show any sign of deformation or rupture; the surface became rough and rippled only a few seconds before the collapse. The landslide, whose triggering point occurred near the top, developed almost entirely in the upper part of the flume. Mobilised soil, therefore, loaded the downslope soil with its weight. Subsequently, after a few minutes, the unremitting slip determined the full collapse of the upper structure, with a subsequent soil liquefaction. The entire sand thickness was mobilised only in the upper part, whilst almost no mobilisation occurred downslope. Surface run-off did not occur. Figure 1(a) shows the collapsed slope at the end of the test.

During the entire process, data from the conventional hydrological instruments were recorded with a frequency of 0.5 Hz, while optical measurements were taken every 12 s for the first 60 minutes and then every 7 s until the end. Noticeably, the sampling time is significantly smaller than that of analogues experiments exploiting BOTDA technique^6,20^. As mentioned above, Rayleigh shift measured at spans A, B, C and D were used to calculate the strain exerted by the slope, while data from span E, not subject to the slope forces, were used to compensate water-induced temperature fluctuations. This has been achieved, as customary, by subtracting the temperature-induced spectral shift measured on span E from the spectral shifts measured on the other spans. However, this temperature compensation had a very limited effect with respect to the spectral shift due to the slope-induced strain, consistent with data provided by electrical temperature probes.

## Results

**Figure 2:**
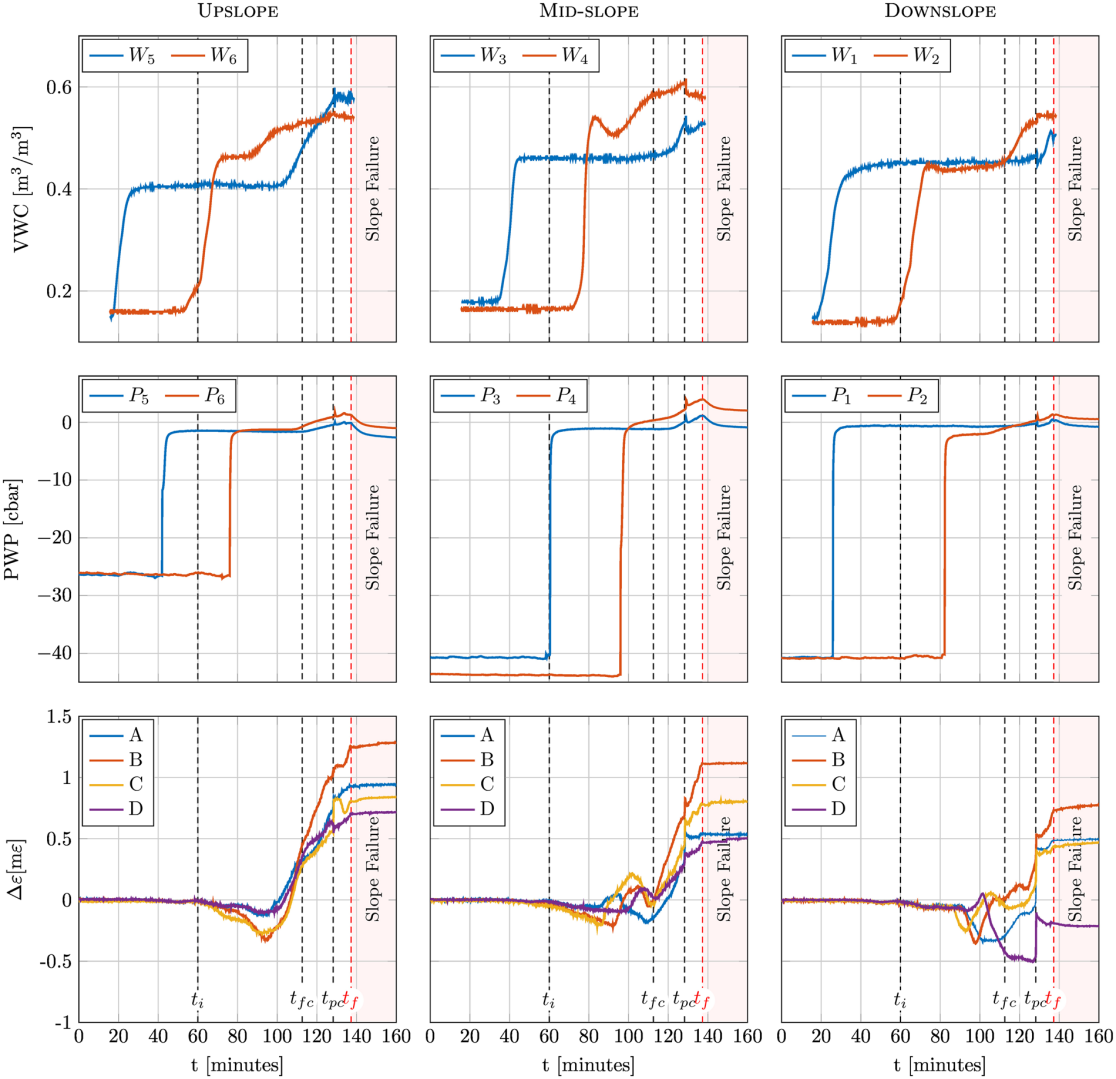
Data recorded vs. time by the sensors at upslope, mid-slope and downslope positions (see Fig. 1(b)). First row: volumetric water content. Second row: pore water pressure. Third row: corresponding local strain measured by the FOS at the different fibre spans as indicated in the legends.

Figure 2 provides in different graphs the trend of monitored data as a function of time from the beginning of rainfall. Measurements are taken at different positions along the slope, so the left-hand, middle and right-hand columns refer to data taken at upslope, mid-slope and downslope positions, respectively (see also Fig. 1(b) for reference). Plots in the top row refer to the volumetric water content (VWC), plots in the second row show the pore water pressure (PWP) and, finally, plots in the bottom row show the strain measured by each of the four spans of fibre cable.

The analysis of the strain evolution in the fibres, compared to visual inspection of the landslide development and other data trends, allowed a tentative identification of some general evolutional phases here listed:

1. *Initial soil-fibre matching phase*. This first phase starts when the water begins to saturate the soil at a depth of the fibre cable, relieving some of the cable-soil friction, thus allowing the cable to release some of the strain accumulated during installation. The phase is recognisable by the development of a very small compressive strain in all the fibres followed by their subsequent oscillatory response.
2. *Fully-coupled soil-fibre phase*. This phase is characterised by a rapid slope deformation, but with no external signs; the slope deformation is detected by a steady temporal increase in the strain along the fibre cable.
3. *Partially-coupled soil-fibre phase*. This phase occurs between the first appearance of tension cracks in the slope and the complete collapse. The phase begins with a sharp strain variation, most likely due to the instantaneous release of energy at the first fissure occurrence, as well as to the initial decoupling between soil and fibre. After the first rupture, the strain varies at a smaller time rate.
4. *Post–collapse uncoupled soil-fibre phase*. After the full collapse, the strain measured by the fibre cable is fairly constant and most likely related to the residual friction between the soil and the cable surface rather than to the actual soil displacements.

The beginning of the first phase can be easily recognised at about *t*_*i*_ = 60 min. As shown in the VWC graphs (first row), the slope is gradually driven to saturation through vertical water flow. The effective stress state at the bottom of the slope is slightly increased by the weight of infiltrating water, but still not affected by an increase in porewater pressure. This phase ends when water reaches the sandy clay bottom, at about *t*_*fc*_ = 110 min approximately. This limit is, however, less evident.

The progressive saturation induces small settlements of the soil slope registered by the FOS, which starts to be strained reaching the full matching of the soil with the cable. The measured strains, more regular at upslope, are dependent on the initial cable configuration at setting up and burying time. Note also that complete saturation of the whole slope seems to occur slightly later with respect to *t*_*i*_, (at $\simeq 80$ min) as shown by the VWC trend of the deeper *W*_4_ probe located at mid-slope.

At about *t*_*fc*_ = 110 min all the cable spans marked a rapid monotonic strain increase; except for span D (see comment below) all the spans showed tensile strains, starting in the upslope section and gradually involving also the mid-slope and downslope sections. At the same time, the PWP sensors record increasing positive porewater pressure, due to the rise of the water table in the sloping sand layer. During this phase, the cable is perfectly coupled with the surrounding soil, and the increase of tensile strain may be interpreted as the development of the downhill slope movement. A closer look at the strain curves reveals that the upslope and mid-slope outer cable spans (namely A and D) experience a slower increase in the strain with respect to the inner spans (B and C). This can be related to the influence of the lateral wall friction, restraining the mass movement, as well as to the retaining effect provided by the small draining wall located at the slope toe. The anomalous behaviour of cable span D in the lower portion of the slope was unluckily caused by an accidental locking with the PWP sensor *P*_4_ which occurred during installation, thus hindering the soil-cable movement. This occurrence was confirmed by the comparison between the Rayleigh shift measured before the test and after the soil deposition (see *Supplementary information* for further details).

The first clear sign of soil detachment is noticed at *t*_*pc*_ = 128 min when a sudden step of strain increase is measured by all cable spans at all positions together with the occurrence of cracks on the slope surface. After *t*_*pc*_, the strain keeps increasing but at a reduced rate, especially in the innermost spans (B and C) at mid-slope and downslope. Also, it is important to observe that all the VWC and PWP probes show a small but noticeable increase in the measured quantities over a very short time interval, probably as a consequence of some significant movements in the soil surrounding the sensors. All the above features, but particularly the change in strain rate, describe a partially-coupled response of the soil-cable system. Moreover, the upslope parts of spans B and C show a significant oscillatory behaviour as a probable consequence of the reaction of the top clamping bars to the force exerted by the sliding soil mass.

The complete failure of the slope clearly occurs at *t*_*f*_ = 137 min; after that, the strain measured by the fibre is almost constant in time. The absence of shear stress may be explained considering two main factors, the first of which is a partial saturation condition of the sand (at *t*_*f*_ the artificial rainfall is stopped, and the sand mass is free to drain through the pervious brick wall at the slope toe) inducing a small suction in the granular matrix. The second factor which must be considered is the occurrence of an arching effect around the cable.

As far as data from the hydrological sensors are concerned, it can be noted that they are consistent with the dynamics of an infiltration front that travels downward but do not provide as many insights into landslide dynamics as the FOS does. A first sharp increase in PWP and VWC can be observed in the uppermost sensors, followed by the deeper ones. This is not sufficient to saturate the soil, as shown by VWC values smaller than the porosity and negative PWP values. A second, more gradual increase is then observed, firstly for the deeper sensors and then for the upper ones, indicating the development of a saturated front (demonstrated by PWP passing from negative to positive values) that moves upward, i.e., the formation of a “perched” water table above the still unsaturated sandy clay layer. The delay of the tensiometer response with respect to the water content probes is in all probability due to the combination of some slight but inevitable spatial variability of the soil properties, as well as to the permeability of the tensiometer porous ceramic cups being smaller than that of the soil^26^. Notably, this behaviour is consistent with a previous experiment and with the numerical simulations performed by Lora *et al*.^22^. The slope failure occurs when the pressure head measured by the deeper tensiometer in mid-slope position is about 2.5 cbar, corresponding to a water table of about 25 cm above the sand-clay interface.

**Figure 3:**
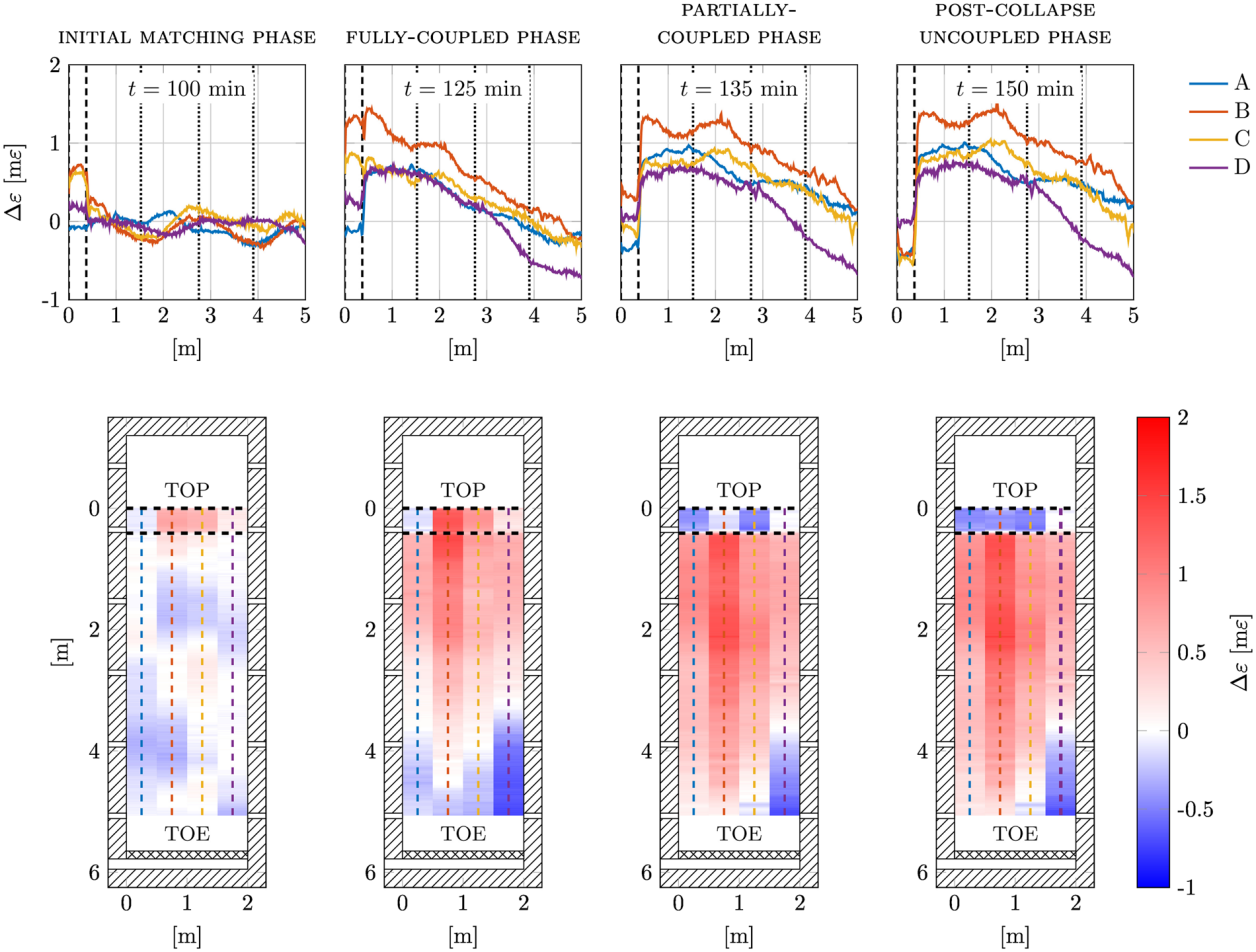
Upper plots: strain field at the four cable spans, namely A, B, C and D, at *t* = 100 min, 120 min, 135 min and 150 min, corresponding to the different regimes of landslide evolution. A dashed vertical line represents the lower clamping bar position. Vertical dotted lines identify up-, mid- and downslope sections where the PWP and VWC sensors are located. Lower plots: corresponding colour maps of the strain field taken at the same instants.

Additional information on the whole strain evolution in the sliding mass can be extrapolated from the spatial variation of the strain along the spans. The upper plots of Fig. 3 show an example of the strain measured along the four spans for each of the phases above; lower plots represent the same data using colour maps. Overall, the figure confirms the classification proposed above and the plot sequence clearly shows the landslide evolution, with the strain increasing with time and decreasing in space from the top to the toe.

Moreover, it can be clearly seen from the colour maps that the strain curves are asymmetric with respect to the centre of the flume; as confirmed by the visual analysis, the collapse started at the left side of the flume, where span B recorded the largest strain.

## Discussion

A physical model reproducing a shallow landslide triggered by rainfall has been fitted with an optical fibre distributed strain sensing system with a spatial resolution of 10 mm. Data provided by this system have been compared to those from conventional sensors installed in a few select positions along the artificial slope. Overall, the optical system has been validated as an unprecedented tool for landslide monitoring and characterisation. Furthermore, its effectiveness and sensitivity are promising for possible use as a reliable early warning and forecasting system.

In that regard, one of the main issues of optical fibre sensing systems in real field conditions is the cable installation. In our opinion, following the approach here described, the optical fibre cable may be embedded in shallow trenches in the ground to monitor the strain field induced in the sensing fibre. To be effective in detecting the triggering of the landslide before any other conventional systems, the optimal position would be near the sliding interface, but also a shallower installation can be effective and more applicable. Indeed, exploiting its high spatial range (up to 2 km of fibre length) the sensor can be deployed all across the potential unstable areas, so as to intersect the boundaries of the landslide even when their location is not known a-priori, and to measure the displacement of the unstable soil mass with respect to the stable ground. Thanks to its high sensitivity and spatial resolution, the sensor might even be capable of detecting the development of small strain within the landslide.

Our experiment also shows that fibre optic sensors are a valuable tool to calibrate early warning systems based on rain thresholds, which are nowadays the most commonly utilised methods. As a matter of fact, the reliability of such early warning systems depends on the accuracy of the hydro-geotechnical models that correlate the amount of rain to the instability of the slope. These must be calibrated by implementing physical models and using *in-situ* and laboratory investigations. Remarkably, physical models are to be preferred over standard laboratory tests as they can better describe infiltration soil properties, especially in quasi-saturated conditions^27^. Finally, we believe that the integration of distributed fibre optic sensors to these physical models would represent a rather slight additional effort if compared to the larger amount of information they provide.

### Data availability

The datasets generated during and/or analysed during the current study are available from the corresponding author on reasonable request.

## Electronic supplementary material

Supplementary information
