# Supplementary material for: Distributed optical fibre sensing for early detection of shallow landslides triggering
Source: Sci Rep. 2017 Oct 31;7:14686. doi: 10.1038/s41598-017-12610-1 (PMC5665914; doi:10.1038/s41598-017-12610-1)
Supplement: Supplementary file 1 — Supplementary information [file 41598_2017_12610_MOESM1_ESM.pdf]

Distributed optical fibre sensing for early detection of shallow landslides triggering

Luca Schenato[1,*], Luca Palmieri[2], Matteo Camporese[3], Silvia Bersan[3], Simonetta Cola[3], Alessandro Pasuto[1], Andrea Galtarossa[2], Paolo Salandin[3], and Paolo Simonini[3]

[1]National Research Council, Research Institute for Geo-Hydrological Protection, Padova, 35131, Italy
[2]University of Padova, Dept. Information Engineering, Padova, 35121, Italy
[3]University of Padova, Dept. of Civil, Environmental and Architectural Engineering,, Padova, 35131, Italy
[*]luca.schenato@cnr.it

Supplementary information

Optical frequency domain reflectometry

The basic scheme of an optical frequency domain reflectometer (OFDR) is shown in Figure 1. The wavelength of the continuous wave light emitted by a coherent laser is linearly swept for some tens of nanometres. The heterodyne beat signal between the light backscattered by the sensing fibre and the local reference allows to measure the distributed Rayleigh scattering along the cable. Comparison between subsequent measurements reveals the Rayleigh spectral shift with resolution in the centimetre range, or even better[1]. This shift is ultimately determined by the strain or temperature field; therefore, by measuring the shift via correlation analysis, the local variation of strain or temperature with respect to an initial condition is retrieved.

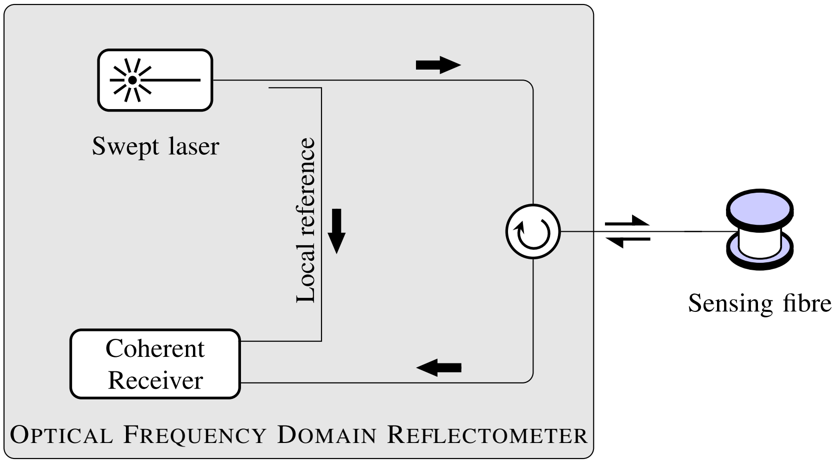

Figure 1. Schematic of an OFDR.

Soil geotechnical characterization

The sand in the upper layer is characterized by a mean particle diameter $d_{50} = 0.2$ mm, effective particle diameter $d_{10} = 0.15$ mm, bulk density ranging from 1169 kg/m^3 (dry) to 1365 kg/m^3 (wet) and porosity of about $n = 0.55$. The bottom sandy clay has fine content $FC(d < 0.075 \text{ mm}) = 67\%$, Atterberg limits $w_L = 0.27\%$ and $w_P = 0.19\%$, critical friction angle $\Phi'_c = 35°$ and residual friction angle $\Phi'_r = 33°$. The hydraulic conductivity of the top layer, as measured by constant head tests on remoulded samples, is about 2×10^{-4} m/s. The saturated hydraulic conductivity of the bottom layer was measured on undisturbed samples both in a falling head permeameter and in a triaxial cell. An the effective stress of 50 kPa, an average conductivity of 5×10^{-9} m/s was found.

Cable specifications and pullout test

The cable used for the experiment (BRUsens© strain v9 by Brugg Kabel AG) is a flexible, mini armored fibre optic strain sensing cable. The cable contains a bending loss insensitive single mode fibre with a multilayer buffer and strain transfer layer with interlocking system, which is embedded inside a low carbon steel tube for protection and hermetic seal (external diameter approx. 0.9 mm). The tube is encapsulated in a polyamide outer sheath with a nominal external diameter of 3.2 mm: the external surface is characterized by 0.7 mm deep indentations with a regular interspace of about 5 mm, to assure a good grip with the materials in which it is buried.

According to the producer, this cable has an elastic stiffness $EA = 47$ kN and is specifically design for distributed strain sensing with strain range up to 1%. Soil movement and ground monitoring, with direct burial in soil, are among the suggested applications.

A series of laboratory pullout tests were performed on this optical fibre cable buried in sand. The sand used in these tests was similar to that used in the slope model (mean particle size $d_{50} = 0.42$ mm, effective particle diameter $d_{10} = 0.18$ mm). The tests were performed inside a $200 \times 40 \times 45$ cm ($L \times W \times D$) tank filled by in-air pluviation with the dried sand. A 1.7 m long cable was horizontally buried at mid height of the tank and additional weigh was applied on the soil top surface to simulate a vertical stress approximately equal to that which acts in the physical model at the base of the sandy layer. The cable was pulled out from a 50 mm circular hole located on the lateral side. Pullout displacements were applied manually by means of a coaxial screw linear actuator. Pullout forces were measured using a load cell anchored between the fibre head and the actuator and

displacements were estimated from the number of rotations applied to the screw actuator. Pullout speed was kept constant at 3.75 mm/minute.

A typical result is shown in Figure 2: the left plot shows the pullout force as a function of the imposed displacement whereas the right graph depicts the distribution of the force along the cable at different displacements pinpointed in left plot and labelled from A to G. Note the reasonably good matching between the pullout force measured through the load cell (blue curve of left plot) with that determined from the cable strain in correspondence to the screw actuator (red curve). The small difference is probably due to some experimental errors inherent to the simplicity of the experimental set up. The overall behaviour shows a brittle response with the force increasing almost linearly up to a maximum of about 150 N and then dropping down towards a relatively stable value. This overall behaviour is very similar to that obtained by Zhang et al.[2], thus confirming the validity of the experiments so far carried out. According to Zhang et al. the whole ascending part of the curve is characterized by an efficient coupling between cable and soil, since the force is linearly distributed along the fibre (curves A, B and C).

At the maximum pullout force, the slope of curve C indicates that the maximum ratio between the mean shear stress developed at the contact soil-cable and the vertical effective stress on the cable plane is equal to about 1, at a strain of about 3200 $\mu\varepsilon$. In this state, the mean friction angle mobilized at the contact is approximately equal to 45°, which is very close to the maximum friction angle of the used sand. This means that the roughness of the polyamide outer sheath provides a good grip with the surrounding soil, as suggested by the producer.

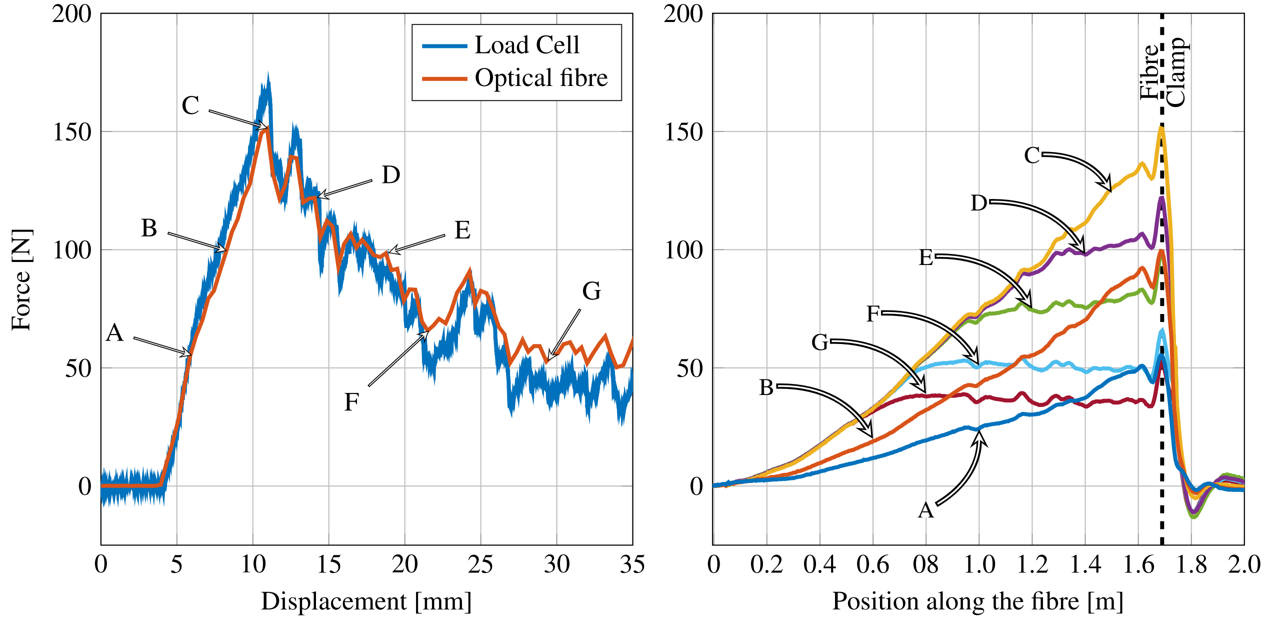

Figure 2. Left plot: pullout force measured by the load cell and calculated from the strain recorded on the fibre cable at the clamping point. Right plot: distributed force exerted along the fibre cable at some selected displacements identified by capital letters in the left plot.

After the pullout force peak (curves from D to G), a non-linear force distribution is observed with the maximum value decreasing as pullout displacement increases. The curves D to G could be subdivided into two parts: the leftmost part overlapped with curve C, gradually reducing its length moving from curves D to G, and the rightmost part characterized by a decreasing slope approaching the minimum pullout force. From this observation it turns out that beyond the peak pullout force, the relative displacement at the contact soil-cable is large enough to reach the critical resistance in the part of cable closest to the clamp.

Strain measured by fibre D at downslope and interaction with clamping bars

The curve corresponding to span D in Figure 1 is the only one showing a stable and marked negative strain at downslope in the interval between t_i and t_{fc}; furthermore, it remains under compression after the collapse, even if the fibre is not clamped at the toe. After further investigation, it has been found that the fibre was accidentally locked by the tensiometer P_4, which pressed the cable against the sandy-clay soil basement. As evidence of that, Figure 3 shows the strain accumulated after the deployment of the upper soil layer followed by the installation of the hydrological sensors. The negative strain peak that can be observed at 2.7 m of span D precisely corresponds to the position at which tensiometer P_4 was installed. This clamping effect significantly

modifies the strain response all along the D, which clearly behaves differently from the other spans.

With reference to Figure 3, the two clamping bars (at 0.0 and 0.4 m from the scarp) are clearly affecting the response of the portions of cable located between them. The strain in the central fibre spans B and C between the two bars initially increases, then, after the collapse, it suddenly decreases, becoming even negative. This could be explained as follows: the lower bar is initially bent under the soil weight, which increases during the test due to the saturation, and as a result, the central fibres in between are elongated. Then, when the slope collapses, the load is released and the bar bounces back to the initial condition (i.e. relative zero-strain state of the fibre) and, after some time, even to a more relaxed state (i.e. negative strain).

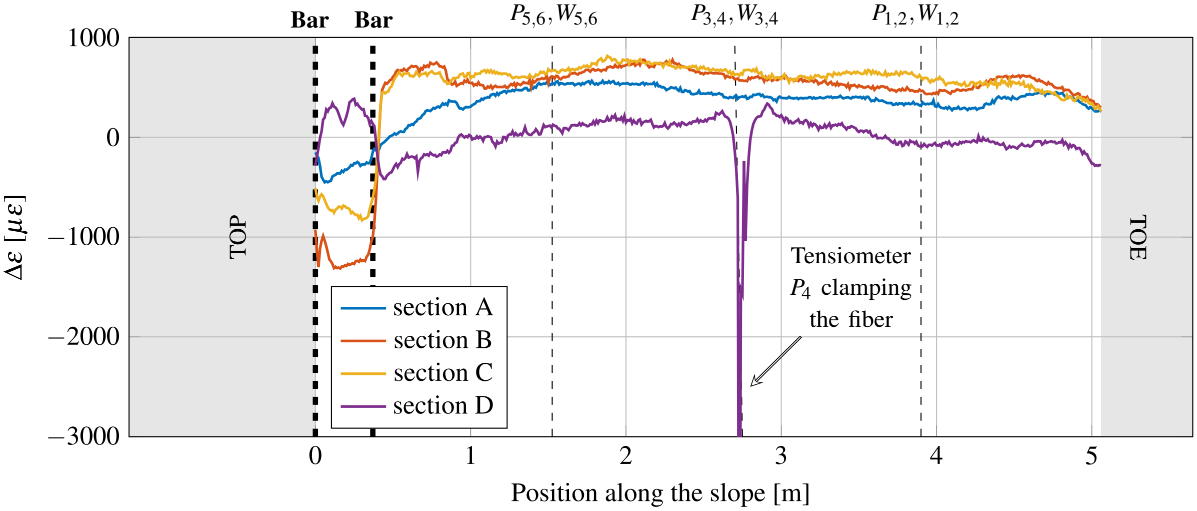

Figure 3. Strain measured at the four fibre spans after the installation of the hydrological sensors in the upper soil layer. Vertical dashed lines locate the positions of clamping bars and hydrological sensors, as indicated by the labels above the plot.

References

1. Palmieri, L. & Schenato, L. Distributed optical fiber sensing based on Rayleigh scattering. *The Open Opt. J.* **7**, 104–127 (2013). DOI 10.2174/1874328501307010104.
2. Zhang, C.-C., Zhu, H.-H., Shi, B. & She, J.-K. Interfacial characterization of soil-embedded optical fiber for ground deformation measurement. *Smart Mater. Struct.* **23**, 095022 (2014). DOI 10.1088/0964-1726/23/9/095022.
